# Knowing Right From Wrong In Mental Arithmetic Judgments: Calibration Of Confidence Predicts The Development Of Accuracy

**DOI:** 10.1371/journal.pone.0098663

**Published:** 2014-07-02

**Authors:** Luke F. Rinne, Michèle M. M. Mazzocco

**Affiliations:** 1 School of Education, Johns Hopkins University, Baltimore, Maryland, United States of America; 2 Institute of Child Development, University of Minnesota, Minneapolis, Minnesota, United States of America; The University of Western Ontario, Canada

## Abstract

Does knowing when mental arithmetic judgments are right—and when they are wrong—lead to more accurate judgments over time? We hypothesize that the successful detection of errors (and avoidance of false alarms) may contribute to the development of mental arithmetic performance. Insight into error detection abilities can be gained by examining the “calibration” of mental arithmetic judgments—that is, the alignment between *confidence* in judgments and the *accuracy* of those judgments. Calibration may be viewed as a measure of metacognitive monitoring ability. We conducted a developmental longitudinal investigation of the relationship between the calibration of children's mental arithmetic judgments and their performance on a mental arithmetic task. Annually between Grades 5 and 8, children completed a problem verification task in which they rapidly judged the accuracy of arithmetic expressions (e.g., 25+50 = 75) and rated their confidence in each judgment. Results showed that calibration was strongly related to concurrent mental arithmetic performance, that calibration continued to develop even as mental arithmetic accuracy approached ceiling, that poor calibration distinguished children with mathematics learning disability from both low and typically achieving children, and that better calibration in Grade 5 predicted larger gains in mental arithmetic accuracy between Grades 5 and 8. We propose that good calibration supports the implementation of cognitive control, leading to long-term improvement in mental arithmetic accuracy. Because mental arithmetic “fluency” is critical for higher-level mathematics competence, calibration of confidence in mental arithmetic judgments may represent a novel and important developmental predictor of future mathematics performance.

## Introduction

The alignment of accuracy and confidence in one's judgment, or “calibration,” has long been of interest to researchers studying judgment and decision-making [1]–[3]. Poor calibration comes in two forms: overconfidence and under-confidence. Recently, several researchers [4], [5] have connected calibration to “metacognition,” a broader concept that encompasses monitoring and regulation of cognitive processes [6]. Although many claim that metacognitive monitoring enhances learning (e.g., [7]), particularly in mathematics [8], others have argued that monitoring is epiphenomenal, having no causal influence on cognition [9].

Here, we report on an empirical longitudinal investigation of children's development of calibration in rapid mental arithmetic judgments. Annually from Grades 5 to 8, children completed an arithmetic problem verification task (PVT) and reported confidence in each judgment on an item-by-item basis. Our aim was to understand the relationship between the development of calibration and the development of mental arithmetic accuracy per se, and to investigate how other cognitive factors (intelligence, executive function, and mathematics achievement) relate to both calibration and accuracy. Such knowledge is important, because accuracy during speeded mental arithmetic (i.e., mental arithmetic “fluency”) is critical for higher-level mathematics competence [10].

Is good calibration merely a *reflection* of computational accuracy, or does good calibration *contribute* to mental arithmetic accuracy? Calibration has been linked to concurrent mathematics performance [11], but the directionality of this association is unclear. We propose that calibration and mental arithmetic accuracy exhibit distinct developmental trajectories, but share a bidirectional relationship. That is, we hypothesize that good calibration is partly a result of high accuracy, but good calibration reciprocally *improves* accuracy by facilitating the implementation of cognitive control.

Good calibration may support cognitive control and contribute to mental arithmetic accuracy in two different ways. First, the ability to discriminate between errors and correct responses as they occur may enable more efficient control over subsequent expenditures of effort and attention. Second, good calibration may enhance cognitive control by accentuating responses to external feedback (e.g., correct answers to arithmetic problems). Feedback is particularly influential following high-confidence errors [12] and low-confidence correct responses [13], but these effects may be contingent on individuals being relatively well calibrated, such that instances of poor calibration are infrequent (and thus deserving of special attention). Poor calibration may dilute effects of feedback on cognitive control, undermining benefits for arithmetic performance. Although we do not directly assess these explanations in the present study, either (or both) could account for the hypothesized effect of calibration on the development of accuracy in mental arithmetic judgments.

The broad notion that good calibration leads to improved cognitive control and better task performance has been proposed previously [14], and experiments testing memory for verbal information have shown that good calibration supports cognitive control by guiding explicit choices about what to study, leading to better recall [15]. Further, interventions aimed at improving calibration have been shown to enhance test performance and/or achievement [16], [17]. However, to our knowledge, no research has investigated the effects of calibration on mental arithmetic performance. Problems above a basic level of complexity require not only explicit recall (i.e., of overlearned solutions to simpler sub-problems), but also the execution of novel computations.

### The Neuroscience Of Error Detection

Neuroscience research on “error detection” is highly relevant to the psychological study of calibration. “Overconfidence” is essentially the failure to detect errors, while “under-confidence” is the *false* detection of errors. Electroencephalography (EEG) studies have shown that following errors, the brain emits event-related potentials (ERPs) referred to as “error related negativity” (ERN), which are widely believed to originate in the anterior cingulate cortex (ACC), a region implicated in conflict monitoring [18]. Although the interpretation of the ERN is controversial, it is often (though not always) associated with error awareness and the engagement of cognitive control (for a review, see [19]). This account is corroborated by studies linking the ERN to slowed response times and improved accuracy following errors (e.g., [20]), which have been reported for a range of activities, including mental arithmetic [21]. Moreover, arithmetic errors produce increased neural activity in the ACC (among other regions), and high mathematical competence is associated with more pronounced post-error activity in the right dorsolateral prefrontal cortex, a region associated with the implementation of cognitive control [22]. Together, these findings support the notion that successful error detection (i.e., good calibration) may influence mental arithmetic performance (at least over the short term) via cognitive control mechanisms.

### The Present Study

We postulate that consistent detection of errors (and avoidance of false alarms) may yield not only short-term changes, but also more enduring improvements in cognitive control during mental arithmetic. The central prediction of our study is that after controlling for the accuracy of arithmetic judgments in Grade 5, better calibration in Grade 5 will be associated with larger improvements in accuracy between Grades 5 and 8. This effect could arise due to better allocation of effort/attention, better utilization of feedback, or both. Although we did not provide feedback on our PVT items, better-calibrated children may have responded more strongly to feedback received in educational settings during the intervening period between Grades 5 and 8.

We also investigate a number of important sub-hypotheses. First, we predict that arithmetic performance will be associated with grade level, mathematics achievement status (low/typical achievement vs. mathematics learning disability), and, consistent with Bol et al. [16], concurrent measures of calibration. However, if calibration reflects unique, causally efficacious metacognitive monitoring abilities, then calibration and accuracy should exhibit distinct developmental profiles and differentiable relationships to cognitive measures. Mental arithmetic, error monitoring (i.e., calibration), and the ERN have all been simultaneously linked to working memory [23], but calibration may also depend on higher-order top-down executive functions, such as response maintenance and cognitive flexibility, which are still developing well into adolescence [24], [25]. Finally, based on previous findings of poor metacognition in children with mathematics learning disabilities [26], [27], we predict that calibration will be uniquely impaired in this subpopulation.

The present study contributes to the growing body of research on developmental predictors of mathematics achievement. Recent work has investigated effects of executive functioning [28], approximate number system (ANS) acuity [29], early number concepts [30], and number system knowledge [31]–[33], and knowledge of fractions [34]. Our aim is to determine whether calibration—a measure of metacognitive monitoring ability—should likewise be viewed as a key predictor of future mathematics achievement. This study elucidates the developmental significance of calibration.

## Materials And Methods

### Ethics Statement

The broader study for which data were collected was reviewed and approved by the Johns Hopkins Medicine Institutional Review Board. All parents provided written consent, and participants provided verbal assent for all study activities.

### Participants

Participants were drawn from a prospective longitudinal study described elsewhere [35], [36] and included students from a socio-economically diverse school district. Participating schools had relatively low rates of mobility and free meal program eligibility (to screen for poverty). Of 445 kindergartners invited to participate, 249 (120 boys) enrolled; most were White (86%). Our primary research question concerned assessments administered during Grades 5–8, so participants were limited to the 190 children still enrolled in the study at Grade 5.

We used scores from a standardized mathematics achievement test to determine participants' mathematics achievement status, based on published criteria [37]. Children who consistently scored above the 25^th^ percentile were classified as having typical mathematics achievement (TA, *n* = 119). Those consistently within the 11^th^–25^th^ percentile were classified as having low achievement (LA, *n* = 26), and those consistently scoring below the 11^th^ percentile were classified as having a mathematics learning disability, or dyscalculia (MLD, *n* = 16). The remaining 29 children were classified as Inconsistent (*n* = 29).

### Measures

#### Mathematics Achievement

The Woodcock Johnson-Revised [38] is a well-established standardized achievement test. We used the Mathematics Calculations subtest (WJ-R Calc), an untimed paper and pencil computation task, to establish mathematics achievement status. Using age-referenced standard scores, we assigned children to TA, LA, or MLD groups, if their scores consistently fell within the 95% confidence interval for the ranges reported previously, or to the Inconsistent group if such criteria were not met.

#### Problem Verification Test

**Figure 1:**
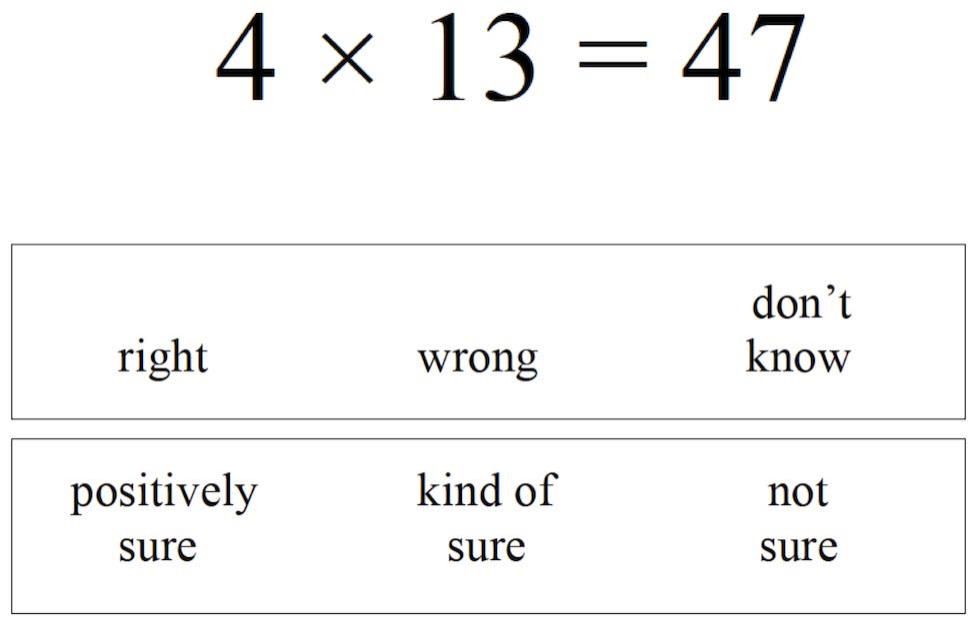
Sample PVT stimulus.

We used a Problem Verification Task (PVT; [39]) to measure children's mental arithmetic accuracy and calibration, our primary outcome variables of interest. The PVT includes 56 two-operand arithmetic expressions (e.g., 220+10 = 230) presented individually, in 48 point font, on the top half of an 8.5″×11″ page. Two rows of prompts appear on the bottom half of each page, providing judgment options for the arithmetic expression presented (“right,” “wrong,” or “don't know”), as well as options for confidence in that judgment (“positively sure,” “kind of sure,” or “not sure”). [The pilot version of the PVT included two levels of confidence (“sure” and “not sure”), but the third level (“kind of sure”) was introduced because many children responded with “kind of sure” even when faced with only those two options.] See Figure 1 for a sample PVT stimulus.

The examiner instructed children to “answer quickly,” coupled with a finger snap to emphasize rapid responding. Practice items ensured that task demands and response options were understood. Although encouraged to provide a right/wrong judgment for all items, children were permitted to report “don't know,” in which case they did not provide a confidence rating. Forty items classified as “easy” tended to involve number combinations that are readily decomposable (e.g., 40−20 = 20) or likely to be overlearned (e.g., 4×4 = 16), whereas the 16 items classified as “hard” tended to have larger problem sizes (e.g., 27+323 = 350) or included incorrect values that are relatively close to the correct values (e.g., 13×4 = 47). Equal ratios of “correct” and “incorrect” solutions were presented across operation and across problem difficulty level.

Individual PVT right/wrong judgments were scored as correct or incorrect, and combinations of confidence rating and correctness were assigned *calibration scores* of 0, 1, or 2, defined as follows: Correct PVT judgments yielded a score of 2 if children were “positively sure” (P), 1 if they were “kind of sure” (KS), and 0 if they were “not sure” (NS). This scale was reversed when PVT judgments were incorrect (i.e., Incorrect/NS = 2, Incorrect/KS = 1, Incorrect/P = 0). Responses of “don't know” (DK) did not receive a calibration score, as no confidence ratings were elicited.

Two different test forms (A and B) were created for use in alternating years. The operands and/or solutions in each mathematical expression appearing in Form A were rearranged to create a comparable expression for Form B (e.g., “50+25 = 75” → “25+50 = 75”; “39÷3 = 16” → “39÷16 = 3”), and the order in which the two halves of the test items were presented was also reversed across forms. Although several additional items were added to the task in Grades 7 and 8, these differed markedly from the 56 core items and were therefore excluded from the present analysis.

#### Wechsler Abbreviated Scale Of Intelligence (wasi)

The WASI [40] is a well-established four-subtest standardized assessment of verbal and nonverbal intelligence, used to estimate overall intelligence in individuals aged 6 to 80 years. We included the age-referenced full-scale intelligence quotient (FSIQ) score as a predictor variable.

#### Contingency Naming Test (cnt)

The CNT [41] is an experimental measure of executive function. This Stroop-like task requires rapid naming of 27 stimuli appearing in a 3×9 array. The stimuli are comprised of ∼1-inch colored geometric shapes, each with a smaller, embedded interior shape (e.g., a blue triangle with an inner square). The “one-attribute” task requires participants to name the stimulus' color (e.g., “blue”) if the interior and outer shapes match, and to name the outer shape (e.g., “triangle”) if the shapes do not match. The more difficult “two-attribute” task requires participants to reverse the one-attribute rule if and only if a backward arrow appears above a stimulus. On both tasks, CNT efficiency is defined as a function of participants' speed and accuracy across all 27 items, as outlined by Anderson et al. [41]:Our analyses included separate predictors corresponding to efficiency scores for the CNT 1-Att. and 2-Att. tasks.

### Data Analysis

Statistical analyses were conducted in four stages. First, we conducted a 4 (Mathematics Achievement Status)×4 (Grade) repeated-measures ANCOVA on PVT judgment accuracy (total number of correct responses), with Grade as the repeated measures variable and mean calibration score as a time-dependent covariate. This analysis served as an omnibus test to establish that development (i.e., grade level), mathematics achievement status, and concurrent calibration scores accounted for significant variance in PVT accuracy.

Second, we created an item-by-item, mixed-effects logistic regression model of PVT accuracy. Here, accuracy is modeled independently of the concurrent calibration score, as calibration scores are only defined post hoc based on observed accuracy and the subsequently elicited confidence rating. The model predicts the odds of PVT correctness (versus error) based on item difficulty, grade level, covariates (e.g., measures of cognitive ability), and random differences between children.

Third, to investigate calibration, we constructed a mixed-effects ordinal probit model of calibration scores. We interpret the probit model according to its latent response formulation [42], in which ordered categorical outcomes (e.g., calibration scores of 0, 1, or 2) indicate whether discrete thresholds in the value of a continuous dependent variable have been surpassed. Here, the latent dependent variable is the individual's overall degree of calibration, which is predicted in terms of PVT judgment accuracy, item difficulty, and grade level, as well as covariates and random effects.

Finally, to investigate whether calibration at one point in time predicts future improvements in the accuracy of mental arithmetic, we created two separate linear regression models that controlled for PVT accuracy in Grade 5 while predicting change in accuracy between Grades 5 and 8. The first model predicts improvement based on average Grade 5 calibration scores on the 0–2 scale described previously. For the second model, we used response frequencies to estimate each child's conditional probabilities of being “positively sure,” “kind of sure,” and “not sure,” given that they were either correct or incorrect. Estimated conditional probabilities were then used as regression predictors.

## Results

### Repeated-Measures Ancova

We used SPSS to conduct a repeated-measures ANCOVA on annual totals of correct PVT responses. There were significant main effects of Mathematics Achievement Status, *F*(3, 172.26) = 17.09, Grade, *F*(3, 491.30) = 8.43, and mean calibration score, *F*(1, 559.42) = 390.16, *p*s<.001. The ANCOVA model explained approximately 60.8% of the variance in PVT accuracy. Test-retest reliability across years is given by the intraclass correlation coefficients for mean calibration score (*r* = .59) and PVT total correct (*r* = .57). Norms for performance accuracy are reported in Table 1.

**Table 1:** Percentage Correct PVT Responses, by Year, Mathematics Achievement Status and Item Difficulty.

	TA			LA			MLD			Inconsistent			All Groups		
Grade	Easy	Hard	Total	Easy	Hard	Total	Easy	Hard	Total	Easy	Hard	Total	Easy	Hard	Total
5	91.3	64.9	83.7	85.6	55.0	76.9	71.3	52.3	65.8	83.3	47.0	72.9	87.6	59.7	79.6
6	91.5	62.6	83.2	87.3	53.1	77.5	71.3	49.5	65.1	89.8	58.2	80.7	89.0	59.7	80.7
7	95.1	71.2	88.3	87.4	56.9	78.7	77.5	50.9	69.9	92.0	59.5	82.7	92.2	66.1	84.7
8	95.7	67.0	87.5	90.8	58.7	81.6	83.6	54.5	75.3	93.8	63.6	85.1	93.7	64.3	85.3
**Total**	93.3	66.4	85.6	87.5	55.8	78.5	75.8	51.9	69.0	89.2	56.3	79.8	90.4	62.3	82.4

**Figure 2:**
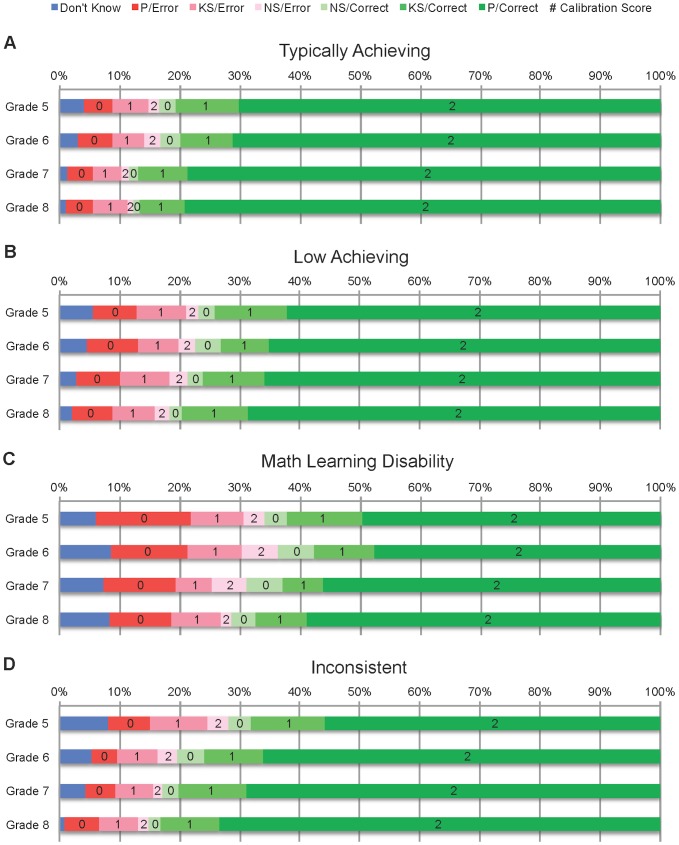
Mean proportion of PVT responses by correctness/confidence, mathematics achievement status, and grade, with calibration scores. For correct (green) and incorrect (red) responses, darker shades represent higher levels of confidence.

Figure 2 shows group mean proportions by year for each combination of PVT accuracy and confidence level. In line with the ANCOVA results, correct responses appear to be strongly associated with higher calibration scores. All groups were confident in most responses, so in any given year, children who answered more items correctly tended to have higher calibration scores. Because generally high confidence levels produce a natural association between high accuracy and good calibration, this analysis does not allow us to discern whether good calibration has any reciprocal effect on PVT accuracy.

Figure 2 also shows the effects of mathematics achievement status and grade level on PVT accuracy. Children with MLD had a greater number of incorrect responses than did their peers, and their corresponding confidence ratings were much more frequently poorly calibrated. For children with MLD, *most* incorrect responses were accompanied by confidence ratings of “positively sure,” yielding a calibration score of 0; in other groups, the rate is considerably lower. From Grades 5 to 8, error rates decreased and calibration scores increased for all four participant groups. It is tempting to conclude from Figure 2 that calibration simply tracks changes in PVT accuracy over time, suggesting that calibration is epiphenomenal—that good calibration is merely a byproduct of high accuracy. However, the aggregation of data across all 56 PVT items and all participant responses in each group may obscure finer-grained relationships between accuracy and calibration. Therefore, we conducted further analyses to examine PVT accuracy and calibration on an item-by-item basis and investigate effects of item difficulty and various cognitive factors. In addition, an item-by-item analysis of calibration allows us to control for the contribution of concurrent PVT accuracy (correctness vs. error). Thus, we can determine whether improvements in calibration over time likely arise due to the development of metacognitive faculties, as opposed to increases in accuracy associated with improving mental arithmetic skills.

### Pvt Accuracy Model

We used Stata's melogit command to create a mixed-effects logistic regression model of correctness (vs. error) in right/wrong PVT judgments. Fixed factors included dummies accounting for gender (Male), item difficulty (Hard Item), effects of development past Grade 5 (Grade: 6, 7, 8), and effects of mathematics difficulty or achievement status (LA, MLD, or Inconsistent). Linear fixed factors included centered WASI scores and centered efficiency scores for the CNT one-attribute (CNT 1-Att.) and two-attribute (CNT 2-Att.) tasks.

Random effects accounted for clustering of data within individuals. Random intercepts capture individual differences in overall error rates. Because individuals may also differ with respect to the effect of item difficulty on accuracy, we added a random coefficient for the item difficulty predictor (Hard Item). A likelihood-ratio (LR) test showed that this significantly improved the fit of the model, χ^2^(2) = 128.36, *p*<.001. The full model is shown in Table 2.

**Table 2:** Logistic Mixed Model of PVT Correctness (vs. Error).

Predictor	*B*	β*_x_*	*SE_B_*	*z*	*p*
Hard Item	−1.957		0.053	−37.100	<0.001
Male	0.460		0.081	5.690	<0.001
WASI	0.006	0.082	0.004	1.650	0.098
CNT 1-Att.	0.175	0.098	0.082	2.140	0.032
CNT 2-Att.	0.161	0.090	0.082	1.960	0.050
Grade 6 (vs. 5)	0.081		0.041	1.970	0.049
Grade 7 (vs. 5)	0.410		0.043	9.530	<0.001
Grade 8 (vs. 5)	0.483		0.044	10.950	<0.001
LA	−0.492		0.124	−3.960	<0.001
MLD	−0.826		0.171	−4.820	<0.001
Inconsistent	−0.371		0.119	−3.110	0.002
Constant	2.268		0.076	29.650	<0.001

*Note.* B = unstandardized regression coefficient; β*_x_* = *x*-standardized regression coefficient.

Converting log-odds to odds ratios (*OR*) aids interpretation of the model. The odds of a correct PVT judgment were 58.4% higher for males compared to females (*OR* = 1.58). Hard items greatly reduced the odds of correctness relative to easy items (*OR* = .15), validating our *a priori* classification. In addition to unstandardized coefficients, Table 2 also gives *x-*standardized coefficients for the three continuous predictors: WASI, CNT 1-Att., and CNT 2-Att. Each had a modest effect on the odds of a correct PVT response. WASI scores (*M* = 109.97, *SD* = 13.41) were a marginally significant predictor of PVT accuracy; a 1-*SD* increase in WASI scores raised the odds of a correct PVT judgment by approximately 8.4% (*OR* = 1.08). Meanwhile, the effect of CNT 1-Att. efficiency scores (*M* = 1.94, *SD* = .57) was significant, with a 1-*SD* increase raising the odds of correctness by 9.4% (*OR* = 1.09). A 1-*SD* increase in CNT 2-Att. scores (*M* = 1.24, *SD* = .58) raised the odds of correctness by 11.4% (*OR* = 1.11); this effect fell just short of significance at the *p*<.05 level.

Across groups, the odds of a correct response in Grade 6 were slightly, but significantly above the Grade 5 baseline (*OR* = 1.08), and the odds of a correct response were dramatically above baseline by Grade 7 (*OR* = 1.51). Gains over baseline appear to level off between Grades 7 and 8, however (*OR* = 1.62). A test of the linear contrast comparing log-odds of correctness for Grades 7 and 8 was not significant, Δ log-odds = .073, *z* = 1.57, *p* = .117. This suggests that by Grade 7, most children (particularly those in the TA group) may be approaching ceiling levels of PVT accuracy for our set of items.

For children with MLD, the odds of making a correct PVT judgment were less than half those of children with TA (*OR* = .44). Smaller decreases in the odds of correctness were also observed for children in the LA (*OR* = .61) and Inconsistent groups (*OR* = .68). Because the number of children in the MLD group (i.e., the number of clusters) was relatively small (*n* = 16), effects of MLD should be interpreted with caution. In order to ascertain whether the small number of clusters in the MLD group affected coefficient estimates or significance tests for other predictors in the model (e.g., item difficulty, grade level, etc.), we removed dummy variables related to mathematics achievement status and re-ran the model. None of the coefficient estimates or significance levels for remaining predictors changed appreciably, with the exception of the coefficient for WASI scores, which increased in magnitude and became significant, *B* = 0.014, β*_x_* = 0.188, *z* = 3.98, *p*<.001, despite being only marginally significant in the full model. However, this heightened effect of WASI scores appears to simply be capturing the effect of the excluded math achievement status variable, which was strongly related to WASI score. A linear contrast testing the relationship between math achievement status and WASI scores (*M_MLD_ = *94.31, *M_LA_* = 104.27, *M_TA_* = 114.88) was highly significant, *F*(1, 186) = 44.08, *p*<.0001. Overall, there is no evidence that the small number of clusters (children) with an MLD classification adversely affected estimates of other effects in the model of PVT accuracy.

### Pvt Calibration Model

We used Stata's GLLAMM command with a scaled ordinal probit link function to construct a mixed-effects partial proportional odds model of calibration scores (0, 1, or 2). The model simultaneously predicts the effects of each independent variable on the probability that a participant surpasses calibration score thresholds at scores of 1 (i.e., a score of 1 or 2 vs. 0) and 2 (i.e., a score of 2 vs. 0 or 1).

Because we interpret calibration as a continuous latent dependent variable, it is natural to assume that error variance is normally distributed, making a probit model more appropriate than a logit model (which assumes a logistic distribution of error variance). The use of a scaled link function allows for a heteroskedastic model by specifying that the log of the standard deviation is equivalent to a linear combination of covariates in the model.

The partial proportional odds model relaxes the proportional odds or “parallel regression” assumption of conventional ordinal probit models. Instead, coefficients are allowed to vary for different thresholds or “cuts” between values of the categorical outcome variable. In the absence of evidence against the null hypothesis that a given coefficient is constant across cuts, we retained the assumption of proportional odds for that predictor.

Fixed factors included all of the predictors from the model of PVT accuracy described previously, along with PVT correctness. Random intercepts accounted for clustering within each participant's set of responses. To build the final model, we started with a “base” random intercept ordinal probit model that maintained assumptions of homogeneous error variance, proportional odds, and fixed coefficient values. We then successively added parameters to loosen assumptions and develop new models, comparing fit at each step using likelihood ratio tests.

A heteroskedastic model significantly improved fit over the base model, χ^2^(7) = 155.34, *p*<.0001. Relaxing the assumption of proportional odds for all predictors further improved the fit, χ^2^(2) = 862.15, *p*<.0001, as did adding a random coefficient for PVT accuracy (Correct), χ^2^(2) = 1936.32, *p*<.0001, followed by a random coefficient for item difficulty (Hard Item), χ^2^(2) = 52.98, *p*<.0001. Once all parameters were added to the model, we conducted Wald tests comparing coefficient values for the first calibration score threshold (score = 1 or 2) to those for the second (score = 2) to determine whether the proportional odds assumption could be retained for some predictors. Coefficient values did not differ significantly for WASI scores, CNT 1-Att. or 2-Att. subscales, or for the LA and Inconsistent mathematics achievement groups (all *p*s>.10). Thus, the proportional odds assumption was retained for these predictors, whose coefficients are identical for both thresholds in the final model shown in Table 3.

**Table 3:** Partial Proportional Odds Model of Calibration Scores.

	Threshold 1: Calibration Score = 1 or 2 (vs. 0)					Threshold 2: Calibration Score = 2 (vs. 0 or 1)				
Predictor	*B*	β*_x_*	*SE_B_*	*z*	*p*	*B*	β*_x_*	*SE_B_*	*z*	*p*
PVT Correct	1.728		0.091	19.020	<0.001	2.006		0.093	21.510	<0.001
Hard Item	−0.289		0.030	−9.530	<0.001	−0.921		0.030	−30.770	<0.001
Male	−0.099		0.042	−2.320	0.020	0.074		0.028	2.610	0.009
WASI†	0.001	0.010	0.001	0.590	0.553	0.001	0.010	0.001	0.590	0.553
CNT-1-Att.†	0.004	0.002	0.028	0.130	0.899	0.004	0.002	0.028	0.130	0.899
CNT-2-Att.†	0.071	0.041	0.027	2.590	0.010	0.071	0.041	0.027	2.590	0.010
Grade 6 (vs. 5)	−0.073		0.031	−2.370	0.018	0.118		0.024	4.960	<0.001
Grade 7 (vs. 5)	0.082		0.033	2.500	0.012	0.180		0.024	7.370	<0.001
Grade 8 (vs. 5)	0.186		0.035	5.390	< 0.001	0.232		0.025	9.190	<0.001
LA†	−0.064		0.041	−1.580	0.114	−0.064		0.041	−1.580	0.114
MLD	−0.017		0.085	0.200	0.841	−0.211		0.058	−3.610	<0.001
Inconsistent†	−0.026		0.039	−0.660	0.508	−0.026		0.039	−0.660	0.508
Constant	2.095		0.052	40.130	<0.001	1.241		0.048	25.720	<0.001

*Note.*

† Proportional odds assumption retained;

β*_x_* = *x-*standardized regression coefficient.

Probit regression coefficients cannot be expressed as odds ratios, making them somewhat more difficult to interpret than logistic regression coefficients. Probit regression coefficients are akin to shifts in the z-score of a standard normal probability distribution. Because the corresponding change in cumulative probability depends on the starting point along the distribution (which in turn depends on the values of all the other predictors in the model), a unit change in a predictor's value can have highly variable effects on the probability of surpassing a particular threshold. On a basic level, however, significant positive and negative coefficients in our model can simply be thought of as respectively indicating increases and decreases in the probability of surpassing a given calibration score threshold.

Consistent with the ANCOVA results described earlier, higher calibration scores were strongly predicted by PVT accuracy. However, hard items significantly reduced calibration scores across both thresholds, particularly the highest threshold, indicating that item difficulty contributes to poorer calibration. Because this model controls for concurrent accuracy (correctness vs. error), the effect of item difficulty on calibration is not simply the result of generally high confidence combined with decreased accuracy for hard items.

Interestingly, males were less likely than females to reach a calibration score of 1 or higher, but were more likely to reach a score of 2. This resulted in large part from females being more likely than males to report being “kind of sure” (KS); males averaged 8.44 KS responses per year, significantly fewer than females' 11.72 KS responses, *t*(188) = −3.04, *p* = .003. Because males tended to express more extreme levels of confidence, they were more likely to have “all or nothing” calibration scores.

As in the previous model of PVT accuracy, Table 3 includes *x-*standardized coefficients for continuous predictors to support comparisons to other effects. There was no significant effect of WASI scores on calibration scores. Meanwhile, higher efficiency scores on the two-attribute CNT subtest predicted higher calibration scores, but scores on the one-attribute subtest did not.

There was a clear developmental trend in calibration scores across the four years of the study. Apart from a small decrease in the probability of reaching the first calibration score threshold during Grade 6, scores increased over time for both the lower and higher thresholds, with increases remaining significant across the last two years of the study (in contrast to PVT accuracy). Tests of linear contrasts comparing Grade 7 and 8 calibration scores indicated that children had significantly higher probabilities in Grade 8 of reaching both the first calibration score threshold, Δ probits = .105, *z* = 2.87, *p* = .004, and the second, Δ probits = .053, *z* = 2.02, *p* = .043. Thus, calibration was still improving at the end of the four-year period studied.

Regarding mathematics achievement, MLD status had a significant effect on calibration, while children with LA, TA, and Inconsistent status did not differ significantly from one another. MLD status did not affect the probability of reaching the first calibration threshold (a score of 1), but children with MLD were significantly less likely than other children to reach the highest threshold (a score of 2). Again, however, these results should be interpreted with caution due to the small number of clusters (children) in the MLD group. As in the previous analysis of PVT accuracy, we re-ran the model without predictors for math achievement status, and only the WASI coefficient changed appreciably. WASI became a significant predictor of the probability of reaching the higher calibration threshold, *B* = .003, β*_x_* = .035, *z* = 2.32, *p* = .020, but not the lower threshold. Thus, the effect of WASI when math achievement status was excluded closely mirrored the effect of MLD status in the full model, providing further evidence that the effect of WASI was simply a marker for the effect of the excluded math achievement status variable.

### Pvt Improvement Model

The final analyses address whether good calibration predicts future improvements in arithmetic performance while controlling for concurrent performance. If the association between calibration scores and PVT accuracy arises simply because good calibration is an epiphenomenon of high accuracy, then earlier calibration scores should not predict future performance when concurrent PVT performance is taken into account. That is, all of the predictive power should reside with measures of PVT accuracy.

To test the alternative hypothesis that calibration *does* contribute to future improvements in PVT accuracy, we built two different ordinary least squares (OLS) linear regression models, each of which predicts improvement in the annual total of correct PVT responses between Grades 5 and 8. Both models included the covariates from previous regression models and the number of correct PVT responses in Grade 5. However, the two models capture the effects of Grade 5 calibration in different ways. The first model (Improvement Model 1) relies on the *average calibration score* (0, 1, or 2) across all 56 PVT items in Grade 5. The second model (Improvement Model 2) relies on separate estimates of conditional probability derived from response frequencies for each possible combination of confidence level and PVT correctness/error. The conditional probability estimates included in the model relate to outcomes yielding sub-optimal calibration scores: *P*(NS | Correct), *P*(P | Error), *P*(KS | Correct), and *P*(KS | Error). Terms for *P*(P | Correct) and *P*(NS | Error) were excluded due to collinearity with other predictors (given the same correctness condition, the probabilities of the three possible confidence outcomes necessarily sum to 1). DK responses (*M_DK_* = 2.66 out of 56) were not modeled, as no confidence levels were provided. The two models are summarized in Table 4. Fully standardized coefficients are given for continuous predictors, while categorical predictors were *y-*standardized but not *x-*standardized.

**Table 4:** Linear Regression Models of PVT Improvement (Grade 8 Sum Correct – Grade 5 Sum Correct).

	Improvement Model 1					Improvement Model 2				
Predictor	*B*	β	*SE_B_*	*t*	*p*	*B*	β	*SE_B_*	*t*	*p*
Male	1.598	0.293†	0.708	2.26	0.025	1.558	0.289†	0.702	2.22	0.028
WASI	0.010	0.025	0.029	0.34	0.732	0.018	0.045	0.031	0.59	0.557
CNT (1-Att.)	0.863	0.090	0.638	1.35	0.178	0.632	0.064	0.659	0.96	0.339
CNT (2-Att.)	−0.346	−0.037	0.653	−0.53	0.597	−0.156	−0.017	0.671	−0.23	0.816
LA	−2.092	−0.384†	1.051	−1.99	0.048	−2.234	−0.384†	1.043	−2.14	0.034
MLD	−3.478	−.639†	1.340	−2.59	0.010	−4.151	−0.767†	1.330	−3.12	0.002
Inconsistent	0.614	0.113†	0.967	0.63	0.527	0.735	0.136†	0.961	0.76	0.446
Gr. 5 Sum Correct	−0.895	−1.017	0.081	−11.00	<0.001	−0.830	−0.926	0.075	−11.14	<0.001
Gr. 5 Mean Calib. Score	6.920	0.269	2.310	3.00	0.003					
*P*(KS | Error)						−0.061	−0.329	0.026	−2.40	0.018
*P*(P | Error)						−0.050	−0.285	0.026	−1.91	0.059
*P*(KS | Correct)						−0.059	−0.138	0.032	−1.85	0.067
*P*(NS | Correct)						−0.188	−0.240	0.064	−2.95	0.004
Constant	31.803		3.482	9.13	<0.001	46.549		4.026	11.56	<0.001

*Note.*

† *y-*standardized only (categorical predictors);

other βs fully standardized (both *x-*standardized and *y-*standardized).

Improvement Models 1 and 2 predicted approximately the same proportions of total variance, 52.8% and 53.3%, respectively. The two models also indicated similar effects of shared covariates. Male gender was associated with greater improvement between Grades 5 and 8, and both LA and MLD status were associated with less improvement. The total number of correct PVT responses in Grade 5 was negatively related to improvement (reflecting a ceiling effect, as children with higher scores in Grade 5 had less room to improve). Nonetheless, Grade 5 calibration scores were strongly predictive of future improvements in performance after controlling for the effect of concurrent PVT performance (partial *r* = .25).

Each of the four conditional probabilities indicating relatively poor calibration were also significantly or marginally significantly related to subsequent improvements in performance. Although the largest effect on improvement scores was associated with a higher probability of being “not sure” of responses that turned out to be correct, this was due to the fact that a higher overall rate of being “not sure” was associated with smaller improvements in PVT accuracy between Grades 5 and 8, regardless of whether PVT judgments were correct or incorrect. In other words, children who were *generally* uncertain of their responses improved less over time. When we replace the *conditional* probability of being “not sure” given a correct PVT response with the *overall* probability of being “not sure,” nearly identical results are obtained (*B* = −.192, β = −.302, *z* = −2.81, *p* = .006), and the values of all other predictors in the model remain essentially unchanged. For the other conditional probabilities in the model, however, substituting the overall probability alters coefficient values considerably and adversely affects the model's performance.

## Discussion

Our study corroborates earlier work on the link between mathematics performance and concurrent calibration, and makes new contributions through a detailed examination of the developmental profiles of mental arithmetic accuracy and calibration, their relationship to one another, and the cognitive factors that predict these profiles. To our knowledge, our study is the first to demonstrate that the calibration of mental arithmetic judgments at one point in time predicts future improvements in arithmetic performance.

Our ANCOVA results are consistent with previous findings by Bol et al. [11] that concurrent calibration is closely related to test performance. However, this ANCOVA cannot clarify the directionality of the relationship between calibration and accuracy, so we conducted additional regression analyses that directly investigated this relationship by modeling student responses on an item-by-item basis. Our logit model of PVT accuracy reveals characteristics that predict arithmetic performance and changes in performance over time. Males had a higher probability of correctness than females, consistent with oft-reported gender differences in mathematics performance (e.g., [43]), but contrary to the results of Lachance and Mazzocco [44], who examined the same sample of children included in the present study and found few and inconsistent gender differences in mathematics achievement during the primary grades. Considering that our study investigated development between Grades 5 and 8, the disparity suggests that gender differences may tend to emerge during the upper elementary and/or middle school years. Alternatively, it could be that gender differences are restricted to tasks similar to the PVT task used in this study, which we did not administer prior to Grade 5.

Intelligence test (WASI) scores were only weakly related to the probability of PVT item correctness, but both the one-attribute and two-attribute subtests of the CNT were strong predictors, highlighting the role of executive functioning in mental arithmetic noted previously by Bull and Scerif [28]. Relative to TA status, MLD status reduced the overall probability of correctness, as did LA and Inconsistent status (albeit to a lesser extent). Finally, PVT accuracy improved over time, but was relatively flat across the last two years of the study (Grades 7 and 8), suggesting that children's development of mental arithmetic ability for items of this difficulty level plateau in middle school.

The probit model of calibration scores showed that after controlling for concurrent PVT correctness, significant effects of cognitive measures and grade level remained, indicating that *calibration is not simply a function of accuracy*. The effect of gender was similar across models of accuracy and calibration, but effects of intelligence (WASI) and one-attribute CNT efficiency were not, as both predicted accuracy, but neither was a significant predictor of calibration scores. Yet, calibration *was* predicted by two-attribute CNT efficiency, suggesting that calibration may be primarily subserved by higher-level top-down executive functions—such as response maintenance and cognitive flexibility. Whereas children with MLD, LA, and Inconsistent achievement were all *less accurate* than children with TA, only children with MLD exhibited *poorer calibration*—as predicted, poor calibration appears to uniquely distinguish children with MLD from children with low achievement. The developmental trend in calibration also differed from that of PVT accuracy. PVT accuracy leveled off after Grade 7, but calibration scores steadily increased across all four years of the study. For arithmetic comparable in difficulty to our PVT items, calibration may take longer to fully develop than mental arithmetic competence. This is consistent with findings that the development of metacognitive abilities occurs relatively late and extends into adolescence and beyond [45].

The fact that calibration scores were significantly lower when children made an error suggests that, to a considerable extent, good calibration *is* a product of high accuracy. Children in all groups were “positive” of their PVT judgments most of the time, less often stating that they were “not sure” or “don't know.” Because confidence levels were generally high, children who got more items correct naturally exhibited better calibration. The tendency to be “positive” in most cases may reflect a general bias toward overconfidence, which has been demonstrated in many other domains (e.g., [46]). In addition, the finding that high item difficulty was associated with poorer calibration exemplifies a common result known in the judgment and decision-making literature as the “hard-easy effect” [47]: Individuals tend to be overconfident for difficult judgments, but well calibrated or sometimes even under-confident for easy judgments. To our knowledge, our study is the first to demonstrate the hard-easy effect on calibration using a mental arithmetic task. Moreover, given that difficulty on the PVT was partly a function of problem size, these results suggest that the “problem size effect”—wherein larger numerical values lead to higher error rates and slower reaction times (for a review, see [48])—may extend to the calibration of mental arithmetic judgments.

Finally, the two regression models of PVT improvement provide converging evidence that good calibration contributes to subsequent increases in mental arithmetic accuracy. When we controlled for Grade 5 PVT performance, better calibration still predicted larger gains in performance between Grades 5 and 8. We speculate that good calibration enables children to more effectively implement cognitive control, leading to better allocation of effort and attention and/or better utilization of feedback. We were unable, however, to directly evaluate this explanation in our current study. Future research should measure or manipulate effort, attention, and responses to feedback in order to better understand the mechanisms behind the effects observed in our study. It is also important to try and replicate the effects of MLD seen here using a sample that includes a larger number of children with MLD. Nonetheless, our results do indicate that good calibration contributes to growth in the accuracy of rapid mental arithmetic judgments. Because mental arithmetic “fluency” is critical for higher-level mathematics competence, the calibration of children's mental arithmetic judgments may represent a unique and important predictor of future mathematics performance.
