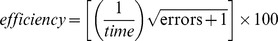# Correction: Knowing Right From Wrong In Mental Arithmetic Judgments: Calibration Of Confidence Predicts The Development Of Accuracy

**DOI:** 10.1371/journal.pone.0109512

**Published:** 2014-09-26

**Authors:** The PLOS ONE Staff

In the HTML version of the article, the equation for CNT efficiency is missing from the paragraph of the Materials and Methods section titled “Contingency Naming Test (cnt).” The equation for CNT efficiency can be viewed below.